# Comparative *in vitro* Assessment of Tooth Color Change under the Influence of Nano Fast Cement and MTA

**DOI:** 10.30476/DENTJODS.2020.85057.1113

**Published:** 2021-03

**Authors:** Fariborz Moazzami, Safoora Sahebi, Sareh Shirzadi, Niloufar Azadeh

**Affiliations:** 1 Dept. of Endodontic, School of Dentistry, Shiraz University of Medical Sciences, Shiraz, Iran; 2 Dept. of Orthodontic, School of Dentistry, Shiraz University of Medical Sciences, Shiraz, Iran

**Keywords:** Tooth discoloration, MTA, Root canal therapy

## Abstract

**Statement of the Problem:**

Tooth color changes followed by treatment with bioceramic materials are always a matter of concern.

**Purpose:**

The aim of the present *in vitro* study was to compare tooth discoloration that occurs in human teeth filled with ProRoot WMTA (DENTSPLY Tulsa Dental Specialties, Tulsa, OK) and those filled with Nano Fast Cement (NFC) over the course of 3 months.

**Materials and Method:**

In this experimental study, Thirty human intact premolars were obtained. The roots of all teeth were removed by a horizontal cut about 2 mm below the cementoenamel junction. The pulp tissues were removed using a barbed broach (Mani, Tokyo, Japan).The teeth were randomly divided to 3 groups (n= 10) including the control (no material), ProRoot WMTA and NFC. The experimental materials were condensed into the crowns and the teeth ends were sealed with light-cure glass ionomer cement (GC Corporation, Tokyo, Japan). The color was assessed at T_BL_ (baseline; after preparation of the cavities but before placement of the materials), T_PO_ (immediately after placement of the filling material and provisional restoration), T_4_ (after 4weeks of storage), and T_12_ (after 12weeks) of storage.

**Results:**

The discoloration was evident in all teeth, immediately (T_PO_) after applying MTA and NFC. The highest ΔΕ was noted in WMTA at 12 weeks, followed by NFC; however, there was no significant difference between the discolorations induced by these two materials.

**Conclusion:**

Similar levels of clinically observable tooth discoloration were detected by using either WMTA or NFC.

## Introduction

Mineral trioxide aggregate (MTA) is a biocompatible material with good sealing and biological properties [ 1 ]. As a root canal treatment material, it has achieved FDA approval since late 20^th^ century [ 2 ]. Due to its exceptional sealing ability and biocompatible behavior, it has been widely implemented in various applications such as apical root-end fillings, perforation repair, direct pulp capping, apexification, and pulp revascularization procedures [ 3 - 8 ]. It is known as the ‘gold standard’ material, despite disadvantages of its use including difficult handling, being expensive, difficult in removing, and discoloration over time [ 9 ]. Concerning the esthetics, the discoloration that occurs produces unsatisfactory results particularly when direct pulp capping or pulpotomy are required in the anterior teeth [ 1 , 10 ]. The first developed MTA was gray and had the potential to cause tooth discoloration. White MTA (WMTA) was developed in order to overcome this disadvantage. The major difference between WMTA and gray MTA is that WMTA contains fewer metal oxides such as Al_2_O_3_, MgO, and FeO, which were assumed to be the main causes of discoloration, but even WMTA has been shown to cause unfavorable tooth discoloration [ 11 - 12 ]. Recently, new calcium silicate base cement named Nano Fast Cement (NFC) was introduced by Shiraz University researchers, which demonstrated favorable properties [ 13 ]. NFC with short setting time and high strength was obtained by modification of MTA material.

Grinding is an important procedure for producing small and ultra -fine particles from solids in powder technology and pharmaceutical industries. In a milling operation, the breakage of initial particles occurs to reduce particle size. The MTA properties vary in different milling time, because the MTA composite have specific mechanical properties in different particle size [ 13 ]. Milling is a top-down particle-forming process, where large drug crystals and excipient are reduced to smaller particles and stabilized in crystalline or semi-disordered crystals with the excipient [ 13 ]. Wet Stirred Media Milling (WSMM) was carried out in time intervals of 5, 10, 15 and 20h. Although decreasing MTA particle size reduces setting time significantly, it shows negative impact on both compressive and flexural strength [ 13 ]. 

The best result for both setting time and strength is obtained at 15 hour milling of material. According to the x-ray diffraction analysis, the MTA was composed primarily of tricalcium silicate, dicalcium silicate, and zirconium dioxide. The results of particle size analysis and scanning electron microscopy images indicated the continued influence of milling on the particle size of MTA. In comparison to MTA, the size of particles was decreased and setting time and handling features were improved [ 13 ]. The tooth discoloration potential of this material however, has not yet been investigated.

Therefore, the aim of the present *in vitro* study was to compare tooth discoloration that occurs in human teeth filled with ProRoot WMTA (DENTSPLY Tulsa Dental Specialties, Tulsa, OK) or NFC over the course of 3 months. Our null hypothesis was that there were no differences in this regard between the two materials. 

## Materials and Method

### Preparation of Extracted Teeth for the Discoloration Experiment

The samples were 30 human premolars extracted for orthodontic purposes. Teeth with clinical and radiographic signs of caries, cracks, restorations, and pathologic discolorations were excluded from the study. The root of each tooth was removed by a horizontal cut about 2 mm below the cementoenamel junction (CEJ), and then the pulp tissue was removed by using a barbed broach (Mani, Tokyo, Japan). Moreover, any organic materials on the surface of the teeth were either physically removed by curettage or by soaking the teeth for 10 minutes in 2.5% sodium hypochlorite solution. The teeth were then washed several times with normal saline and stored at 4⁰C until the experiment time.

The teeth were randomly divided to 3 groups (n= 10 teeth per group) according to the material used to fill their pulp chamber namely control (no material), ProRoot WMTA, and NFC. The materials were prepared as instructed by the manufacturer and then used to retrofill the teeth to the level of the CEJ in the pulp chamber. After the early setting phase, light-cure glass ionomer cement was used to seal the lower area (GC Corporation, Tokyo, Japan). In the control group, teeth were only sealed with light-cure glass ionomer cement (GC Corporation, Tokyo, Japan).Immediate postoperative color measurements were obtained, and specimens were stored in a dark environment (100% humidity, 37⁰C) with normal atmospheric gas levels for the subsequent color measurement procedures.

**Figure 1:**
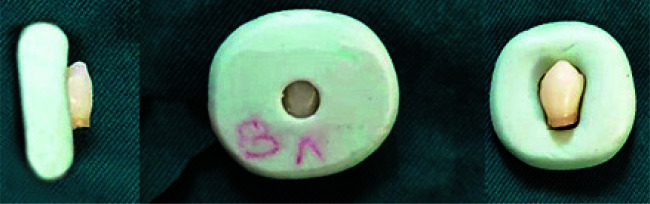
The putty mold used in this study for holding the test teeth to allow consistent measurement of L*, a* and b* values

Molds were fabricated for each tooth using rubber impression material (Aquasil Soft Putty, Dentsply) (Figure 1). Holes with diameters of about 6 mm were created in the molds using a biopsy punch (KAI medical, Tokyo, Japan) so that the extent of discoloration could be measured at the same site. The hole was located so that it revealed an area between the midbuccal third and cervical third to facilitate observation of any MTA-induced internal discoloration.

### Measurement of Tooth Color

VITA Easyshade Compact device (Vita Zahnfabrik, Bad Sackingen, Germany) was used to measure tooth color at the following time points: T_BL_ (baseline; after preparation of the cavities but before placement of the materials), T_PO_ (immediately after placement of the filling material and provisional restoration), T_4_ (after 4 weeks of storage), and T_12_ (after 3months) of storage. The color measurement was made in triplicate for all experimental groups using the L*a*b* value, where L* is the light intensity, a* is the red-green parameter, and b* is the yellow-blue parameter. Using these L*a*b* values, the differences between the color measured at the initial time point (baseline) and those measured at the various time points (∆E) were calculated using following equation: ∆E=[(∆L)^2^+(∆a)^2^+(∆b)^2^]^1/2^

### Statistical Analyses

All statistical analyses for differences in color changes among the products between the initial time point and the other time points after the initiation of light treatment were conducted using SPSS software (versions 21.0; SPSS IBM, Armonk, NY). Nonparametric One-Way ANOVA (*p*< .05) was performed, and the Dunn's test (*p*< .05) was used as a post hoc test.

## Results

**Figure 2:**
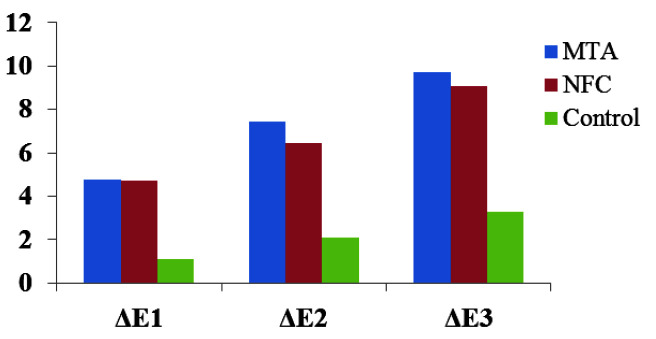
Mean (SD) ΔΕ values of groups 1–3 in all experimental periods

Discoloration was evident in all teeth, immediately (T_PO_) after applying MTA and NFC. MTA and NFC induced significantly more severe discoloration compared to the control group (*p*<0.05). For these groups, the mean *ΔE* values increased until the third month during the period of investigation. The highest ΔΕ was noted in WMTA at 3 months, followed by NFC, but there was no significant difference between the discolorations induced by these two materials. Total color change induced by MTA and NFC was higher than the perceptibly threshold (3.3) at all time periods (Table 1, Figure 2).

**Table 1:** Mean (SD) ΔΕ values of groups 1-3 in all experimental periods

Group	*ΔE*1	*ΔE*2	*ΔE*3
MTA	4.7640(2.32706)	7.4420(2.51670)	9.7280(2.80679)
NFC	4.7000(2.02409)	6.4230(1.41866)	9.0590(1.70196)
Control	1.1080(.55445)	2.0660(.92199)	3.2740(.63535)

## Discussion

Patients are commonly concerned by the unaesthetic appearance of teeth caused by certain dental materials after restorative procedures [ 1 ]. MTA is commonly used endodontic cement, which has been shown to cause tooth discoloration [ 2 , 14 ].

Color measurement studies implement several devices such as colorimeter [ 15 ] and spectrophotometer [ 2 , 10 , 12 , 16 - 19 ]. High resolution digital photographs and color analysis by Photoshop software can also be used for this purpose [ 18 , 20 ]. Dozic *et al*. [ 21 ] concluded that Easy Shade spectrophotometer and digital camera are the most reliable tools for color assessment. In the current study, a spectrophotometer was used. Spectrophotometer and spectroradiometer measure the light reflection index in the visible spectrum. As the most accurate and commonly used system for color measurement, the CIE L*a*b* system was used in the current study. This system quantifies the change in color parameters. Color change is clinically perceptible in values > 3.3 [ 18 ]. Our study investigated tooth discoloration behavior of a newly introduced root filling material, NFC and the results indicated that this material as well as WMTA caused significant color changes immediately after placement with persistent discoloration in the second and third time periods. Although the highest ΔΕ was noted in WMTA, there was no significant difference between the discoloration induced by WMTA and NFC.

Similar to our study, most studies such as Ioannidis *et al*. [ 1 ], Ramos *et al*. [ 9 ] and Kang *et al*. [ 2 ] showed that discoloration increases with time after using MTA. The discoloration observed immediately after exposure to MTA may be due to the change in dental transparency, it is expected that it will resolve with removal of the material. 

In contrast to the results of our study, most studies indicated that significant color changes occur, after more than four weeks [ 22 ]. For instance, Ramos *et al*. [ 9 ] detected clinically perceptible crown discoloration by WMTA in the 6thweek of their experiment. Although premolars were used in this study like our study, colorimeter was implemented to detect color change, which is different from our study; this may explain the different results yielded by two studies.

In addition, Ioannidis *et al*. [ 1 ] demonstrated that GMTA and WMTA induced clinically perceptible crown color change from the first and third months of application, respectively. This different outcome from our study may be due to the fact that they have evaluated color change in 3rd mandibular molars while we tested premolars and the difference in structural characteristics of these teeth may have resulted in different discoloration behaviors. In their study, GMTA resulted in greater discoloration than WMTA at all experimental time periods. 

Our results however, are consistent with those presented by Esmaeili *et al*. [ 22 ] and Meetu *et al*. [ 23 ], who found that significant color changes occur only one week after placement of WMTA.

The same formulation of MTA resulted in variable amounts of color change in different studies [ 24 - 25 ]. The time duration between exposure of teeth to MTA and detection of discoloration also differed between teeth. This inconsistency between studies could be the result of different thicknesses of the remaining tooth structure, variety in colorimetric methods, and dissimilar methods of material application [ 24 - 25 ].

Since NFC contains nano-sized particles, it may be anticipated that it would penetrate deeper in dentinal tubules and cause more severe discolorations than WMTA; in this study however, our null hypothesis was proved and no difference was observed between discolorations of the two experimental materials.

NFC has recently been introduced in literature and has not been investigated with regard to its effect on tooth color; therefore, the result of our study could not be compared with those of similar studies.

In contrast to some researchers who used composite resin to seal the root end [ 26 - 27 ]; glass ionomer cement was used in the current study, so that it could better seal dentinal tubules. Due to the bond of carboxylic ions to calcium ions of hydroxyl apatite and chemical bond to tooth structure, this cement was also implemented in similar studies [ 28 ].

Our study included an adequate sample size. In addition, in order to compare the discoloration potential of teeth more accurately, we used extracted sound human teeth similar to many previous studies [ 2 , 15 , 20 ].

Despite these efforts to simulate the clinical conditions, the *in vitro* design is subject to imperfections. Hence, generalization of the results to the clinical setting must be done with caution. Future clinical studies are required to better elucidate the discoloration potential of WMTA and NFC in the oral environment [ 14 ].

## Conclusion

Clinically perceptible crown discoloration was detected immediately after applying WMTA and NFC. There was a similar level of clinically observable tooth discoloration detected using either WMTA or NFC and crown discoloration demonstrated an increasing pattern with time. Application of both biomaterials should be considered with high level of caution, especially in the esthetic zones.
